# Progress in Surgery

**Published:** 1898-08-27

**Authors:** 


### Progress in Surgery.

### GENITO-URINARY SURGERY.

**The Bacteriology of Cystitis** has been investigated by Dr. Hugh Young by means of cultures from fluid obtained by suprapubic puncture of the bladder.[1] Of 18 cases of acute cystitis he found the staphylococcus pyogenes albus in eight, generally after the use of instruments. He thinks the bacillus coli is more associated with chronic cystitis, and seems then to drive all the other micro-organisms away. It is remarkable that he found five cases sterile even after the greatest care.

**Vesical Calculus.** — Dr. P. J. Freyer gives his experience of his last 100 cases of vesical calculus.[2] He states that he has practically abandoned all other operations for stone in favour of Bigelow's litholapaxy. In the 100 cases he only twice cut for stone. It is essential to success to have proper instruments, and advisable to have the lithotrite fully fenestrated, and the evacuator as perfect as possible. With practice the operation can be rapidly performed. Dr. Freyer has performed litholapaxy on a child aged one and a half years, and on an old man of 85. In females, litholapaxy is eminently successful, and does away with permanent incontinence which follows rapid dilatation of the urethra. The period of convalescence is from two to 20 days. In England vesical calculus is frequently complicated by enlargement of the prostate. Freyer is strongly opposed to any routine suprapubic operation.

Of three cases exhibited by Mr. Lynn Thomas two were of unusual weight, one being nearly six and a half and the other seven ounces in weight. They were removed by the suprapubic operation.[3]

**Cysts of the Urachus.**—A paper was recently read by Mr. Alban Doran on this subject.[4] They occur between umbilicus and pubes, and between the peritoneum and abdominal parietes. Cases have been described as "large urachal cysts" which have really been encysted dropsies associated with tuberculous peritonitis. In their treatment incision and drainage are usually applicable. If they be dissected out, one should not leave a large area of peritoneum behind, as it would be apt to slough, and one should carefully inspect the relations to the abdominal viscera and to the bladder below. Mr. Bryant referred to two cases under his care.

Rotter recently operated on a case of cyst of the urachus associated with cancer of the bladder, which was detected by the cystoscope. Rotter removed the whole cyst, together with part of the fundus of the bladder.[5]

**Tuberculosis of the Bladder.**—Failing constitutional remedy, Mr. Mansell Moullin holds that suprapubic cystotomy is the only chance of success, and should be done early. Early diagnosis is now possible by the cystoscope.[6] Others are disposed to give up the surgical treatment of this condition as unsatisfactory.

**Vesical Irrigation.**—This is now very largely used in America for the treatment of gonorrhœal urethritis and cystitis, a copious irrigation of permanganate of potassium being generally recommended. For intravesical irrigation it is necessary to raise this vessel to a height of from six to eight feet above the level of the patient's bladder. The anterior urethra should first be thoroughly flushed with a copious irrigation. Various preparations of silver have been employed for irrigation and injection, and the latest—protargol, a combination of silver with albumen—appears to be very effectual.

Fürst has found protargol to be the safest and best application for gonorrhœa in women.[7] He says that a cure can be effected in three weeks, but only with very thorough treatment in the absence of gonorrhœal salpingitis.

**Enlarged Prostate.**—According to Alexis Thomson, operative treatment is only indicated when catheter life is failing. In advanced septic cases suprapubic drainage is indicated, but in non-septic cases suprapubic prostotectomy is the ideal treatment. If unsuitable for the above, then double castration is advisable, although less reliable.[8] Although castration is regarded as more certain than resection of the vas deferens, yet Mr. Hoffman thinks that the latter is not notably inferior to the former in certainty of results.[9] It seems to be advisable to remove the surrounding nerve fibres when resecting the vas.

**Prostatic Calculi.**—Mr. Golding Bird has published an interesting case of this condition, in which he removed 130 stones from the left lobe of the prostate. The stones are represented upon a photograph, and a skiagram shows the condition before operation.[10]

**Urethral Stricture.**—Urethrectomy is now performed with success for cicatricial and infected masses in the region of the urethra, as in old perineal fistulæ, and in cases in which there is cicatricial stenosis of the urethra. All cicatricial tissue should be removed as well as any infected tissue in the neighbourhood. When sutured over a catheter a new epithileum-lined canal is formed. Resection of a part of the circumference of the urethra is very successful.[11]

Reginald Harrison advises both internal and external urethrotomy combined, in certain cases of stricture which are liable to relapse. The mere fact of perineal drainage for a few days seems to exercise a salutary influence on the process of repair and permanent restoration of calibre.[12]

**Prepuce Cysts.**—These are rare, but interesting. The literature of the subject is considered by Dr. G. H. Edington.[13] He also described a case of his own having the characters of a dermoid. Cysts of the prepuce are of four classes: (1) Sebaceous cysts arising from Tyson's glands on the inner aspect of the prepuce or from glands on the outer aspect. (2) Mucous cysts, which have mucoid contents. (3) Congenital cysts of the raphé. These are allied to developmental inclusion dermoids. (4) Implantation cysts, due to traumatism. These are apparently more common in Jews.

**Chancres.**—Unna (Hamburg) practices either total excision of the chancre after disinfection, with union of the edges to avoid a scar, or else superficial section and treatment with nitrate of silver, &c., to hasten the healing process.[14]

**Phimosis.**—Dr. Daggett suggests an ingenious modification of the slit method of treating this condition. He makes a ventral slit at the prepucial orifice and a dorsal slit over the corona glandis. When the foreskin is drawn over the glans the incisions become transverse, and are united in that position.[15]

Symposthion or adhesions of prepuce to glans is apt to occur in phimosis, and it may be necessary to deal with the condition before gonorrhœa is successfully cured.[16]

**Syphilis.**—Various lesions have lately been described. Heuzard speaks of syphilitic phlebitis, an acute form in the secondary stage, and a chronic form in the tertiary.[17] Green describes four varieties of syphilitic affections of the kidneys—(*a*) parenchymatous, (*b*) interstitial nephritis, (*c*) gummata, (*d*) amyloid degeneration.

Dr. George Ogilvie confirms the impression generally held as to the inadequacy of the present system of treatment of the disease in India.[18]

[1] New York Med. Rec., May 21, 1898. [2] Lancet, May 14, 1898. [3] Lancet, May 28, 1898. [4] Lancet, May 28, 1898. [5] Epit. Brit. Med. Jour., May 28, 1898. [6] Brit. Med. Jour., May 14, 1898. [7] Therapeutic Monatshefte, April, 1898. [8] Brit. Med. Jour., June 11, 1898. [9] Beiträge z. Klin. Chir. xix., 3. [10] Brit. Med. Jour., July 2, 1898. [11] Med. Rec., May 7, 1898. [12] Lancet, June 11, 1898. [13] Glasgow Med. Jour., June, 1898. [14] La Semaine Medicale, Mar. 23, 1898. [15] Med. Rec., May 7, 1898. [16] Deutsch. (Wien. Med. Presse, 1898, No. 19). [17] Thèse de Paris, 1893, No. 179. [18] Brit. Jour. of Dermatology, July, 1898.

### SURGERY OF THE SPINE.

**Hæmato-myelia from Gunshot Wounds of the Spine.**—In reporting two cases of gunshot wounds implicating the spinal column, and giving rise to symptoms of cord lesion, Cushing[1] discusses this question in some detail. In both of his cases the missile lodged in the body of a vertebra, and was located by means of the X-rays. Although the cord was not injured directly in either case, each presented symptoms resembling Brown-Séquard paralysis. Recovery without operation followed in both cases, but there remained a residuum of symptoms resembling syringo-myelia.

The first case, that of a young woman, 27 years of age, who was shot at close range, is described at great length and with much care. The ball entered at the anterior border of the right trapezius at the level of the cricoid cartilage, and lodged in the body of the seventh cervical vertebra. Right-sided hemiplegia below the level of the deltoid, biceps, and supinator longus, with pain, supervened.

Among other interesting points in the progress of the case, the writer mentions that it is not uncommon in cases of cervical lesion to meet with an elevation of temperature of two deg., followed by a chill and further rise up to 104 or 105 deg. This usually means, as it did in his own case, the onset of a basal pneumonia, with consolidation on the side of the lesion. It may be connected with the intercostal paralysis. The pyrexia remained for about 28 days. During the next month great progress was made, but a relapse followed, and for another month the patient was much distressed. Slow but steady improvement ensued during the following six months.

Of the subjective symptoms, the most outstanding were agonising numbness and tingling; severe pruritis; burning sensations and lancinating pains; following in this sequence, first in the left and then in the right arm. Four days after injury, severe girdle pain was first complained of. This recurred at the end of the second month, and was most severe on the paralysed (right) side. Other sensory symptoms, such as icy-cold feet, spasticity of the right leg cutting pains in the arm, &c., were present at different times.

The reflexes at first were present, especially on the non-paralysed side (left). Forty-eight hours later they became exaggerated. Four days later they were lost, and after three more days reappeared and gradually increased till they became much exaggerated. A similar sequence of reflex phenomena was observed by Kocher in his case of hemilesion from stab high in the cervical cord.

Motor disturbances were more transitory on the left side than on the right. Eventually all motion was restored, except in the dorsal flexers of the right foot. The nutrition of the patient was much interfered with, although food was well taken. Special nutritional disturbances were observed in the right hand in the form of swelling, tense shiny condition of the skin, furrows on the nails, and flushing. Such disturbances, according to J. K. Mitchell, only occur in partial lesions of the cord, and are due to loss of trophic control.

Discussing the nature of the spinal lesion, the writer cites evidence in favour of the view that the mere passage of a bullet near the spine may produce symptoms of concussion, but inclines to the opinion that this means a degree of hæmato-myelia with minute hæmorrhages into the cord. He agrees with those authorities who believe that traumatic hæmato-myelia is commoner than is generally supposed, whereas the meningeal form of hæmorrhage is comparatively rare.

The diagnosis between meningeal hæmorrhage and hæmorrhage into the substance of the cord is often exceedingly difficult. In the former irritative phenomena predominate; acute and immediate spinal pain is constant; and radiating pains along the line of distribution of the nerve roots involved is characteristic. In the latter (intra medullary) form paralytic symptoms of motility and sensibility are most in evidence, and for anatomical and physiological reasons assume the Brown-Séquard type. Combined lesions may exist.

An important observation of Thorburn's is quoted and emphasised, namely, that traumatic hæmato-myelia is very rarely met with except in cases of lesion of the cervical spine. This the writer explains on the assumption that the large amount of grey matter at the lower cervical enlargement favours blood effusion. He even goes the length of suggesting that there is an artery which, like Charcot's artery of cerebral hæmorrhage, is the seat of election for such extravasations. The finding of cyst-like cavities in the cord long after such injuries lends support to these views, as well as the resemblance of such cases to syringo-myelia. These findings discourage surgical intervention in this class of cases. The site and extent of the lesion are more easily diagnosed, the distribution of cord segments being fairly well established.

The following are the conclusions arrived at: (1) Paralytic symptoms following traumatism in the cervical region, when there is no resultant spinal deformity or laceration of the cord, are in the majority of cases due to hæmorrhage into the substance of the cord; (2) for the hæmorrhage there seems to be a site of predilection in the lower part of the cervical enlargement; (3) the hæmorrhage, as a rule occurring primarily on one side, leads to symptoms of a Brown-Séquard type of paralysis; (4) the deep reflexes on the side of hemilesion may be retained for a time, then disappear, and finally return to become exaggerated; (5) the hæmorrhage, being primarily into the grey matter, and in its resolution often leading to cyst-like formation, is productive in many cases of a symptom complex, quite like that of syringo-myelia; (6) the immediate prognosis in this type of hæmato-myelia is good without operative interference, even in the cases of gunshot wounds, when they are uncomplicated by sepsis. The second case, quite a recent one, is described in less detail, but bears out the conclusions arrived at from a study of the first.

A case of spinal fracture at the level of the ninth or tenth dorsal vertebra is reported by Lyons.[2] The paralytic symptoms did not come on for some hours after the accident, and were therefore probably due to slow effusion of blood. The right leg was most paralysed, and physical signs appeared on the right side of the chest, with pyrexia and a blood-stained expectoration. There was no girdle pain. Both knee reflexes were lost.

[1] Amer. Jour. Med. Sci., 1898. [2] Lancet, June 25, 1898.

